# Seismic surveys near gray whale feeding areas off Sakhalin Island, Russia: assessing impact and mitigation effectiveness

**DOI:** 10.1007/s10661-022-10016-9

**Published:** 2022-10-18

**Authors:** Lisanne Aerts, Michael R. Jenkerson, Vladimir E. Nechayuk, Glenn Gailey, Roberto Racca, Arny L. Blanchard, Lisa K. Schwarz, H. Rodger Melton

**Affiliations:** 1 LAMA Ecological, Anchorage, AK 99502 USA; 2 ExxonMobil Exploration Company, Spring, TX 77389 USA; 3 Cetacean EcoSystem Research, Lacey, WA 98512 USA; 4 JASCO Applied Sciences, Victoria, BC V8Z 7X8 Canada; 5 Blanchard Ecological, North Pole, Alaska, AK 99705 USA; 6 Ocean Sciences and Institute of Marine Sciences, University of California, Santa Cruz, CA 95060 USA

**Keywords:** Russia, Sakhalin Island, *Eschrichtius robustus*, Seismic survey, Mitigation effectiveness, Bioenergetics

## Abstract

In 2015, two oil and gas companies conducted seismic surveys along the northeast coast of Sakhalin Island, Russia, near western gray whale (*Eschrichtius robustus*) feeding areas. This population of whales was listed as Critically Endangered at the time of the operations described here but has been reclassified as Endangered since 2018. The number and duration of the 2015 seismic surveys surpassed the level of previous seismic survey activity in this area, elevating concerns regarding disturbance of feeding gray whales and the potential for auditory injury. Exxon Neftegas Limited (ENL) developed a mitigation approach to address these concerns and, more importantly, implemented a comprehensive data collection strategy to assess the effectiveness of this approach. The mitigation approach prioritized completion of the seismic surveys closest to the nearshore feeding area as early in the season as possible, when fewer gray whales would be present. This was accomplished by increasing operational efficiency through the use of multiple seismic vessels and by establishing zones with specific seasonal criteria determining when air gun shutdowns would be implemented. These zones and seasonal criteria were based on pre-season modeled acoustic footprints of the air gun array and on gray whale distribution data collected over the previous 10 years. Real-time acoustic and whale sighting data were instrumental in the implementation of air gun shutdowns. The mitigation effectiveness of these shutdowns was assessed through analyzing short-term behavioral responses and shifts in gray whale distribution due to sound exposure. The overall mitigation strategy of an early survey completion was assessed through bioenergetics models that predict how reduced foraging activity might affect gray whale reproduction and maternal survival. This assessment relied on a total of 17 shore-based and 5 vessel-based teams collecting behavior, distribution, photo-identification, prey, and acoustic data. This paper describes the mitigation approach, the implementation of mitigation measures using real-time acoustic and gray whale location data, and the strategy to assess impacts and mitigation effectiveness.

## Introduction

**Fig. 1:**
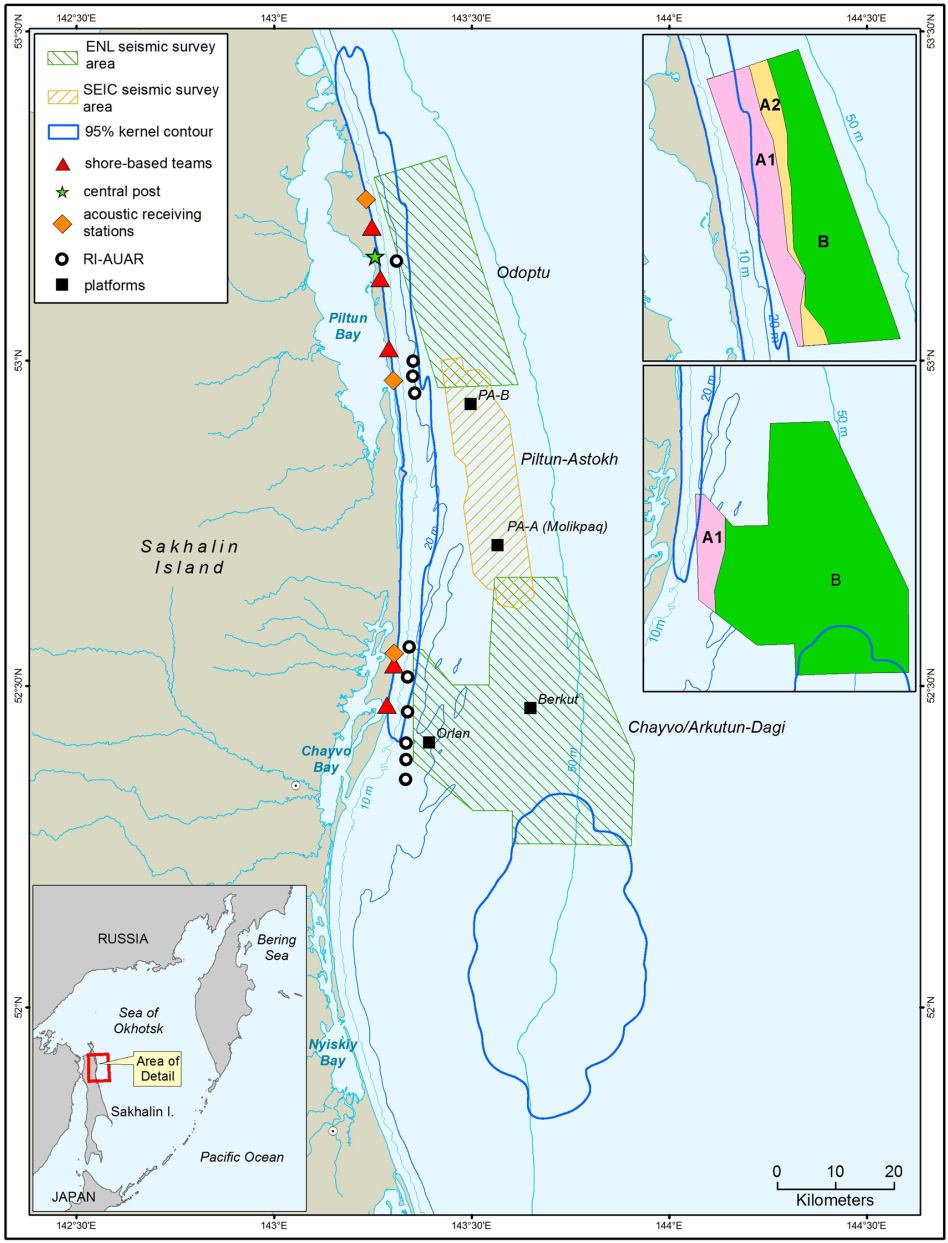
Overview of the 2015 seismic survey areas and their position relative to the 95% kernel contours of the nearshore and offshore feeding areas. The Chayvo and Arkutun Dagi seismic surveys were adjacent to each other and are therefore shown as one area. The insets on the upper right show the mitigation zones for the seismic survey areas. Shore-based acoustic stations received data from the Autonomous Underwater Acoustic Recorders with VHF and Iridium data transmission capability (IR-AUAR) (Rutenko et al., 2022a)

In the 1970s and 1980s, a small number of gray whales (*Eschrichtius robustus*) were observed feeding in the nearshore waters off the northeast coast of Sakhalin Island, Russia (Blokhin et al., 1985; Brownell & Chun, 1977). Prior to these observations, this population, referred to as the Korean-Okhotsk or western gray whale population, was believed to be extinct due to commercial whaling activities (Bowen, 1974). Little was known about the life history and ecology of these whales other than that most individuals were observed to return to what became known as the “(Piltun) nearshore feeding area” each year (Weller et al., 1999). Based on available information about post-whaling recovery of this population and the low numbers of reproductive females, the International Union for the Conservation of Nature (IUCN) listed the western gray whale as Critically Endangered in 2000 (Hilton-Taylor, 2000). In 2001 and in subsequent years, relatively large numbers of gray whales were observed offshore of Chayvo Bay (Meier et al., 2007) in what became known as the “offshore feeding area” (Fig. 1). In 2018, after completion of the work described here, the IUCN re-classified the western gray whale as Endangered based on data that included gray whales observed not only off Sakhalin but also off Kamchatka (Cooke et al., 2018).

Oil and gas reserves were discovered near the northeast Sakhalin coast starting in 1977. To ensure maximum economic benefits from development projects, the Russian Federation instituted production sharing agreements with Exxon Neftegas Limited (ENL) and Sakhalin Energy Investment Company (SEIC) in 1995, both of which had shown interest in extracting these offshore reserves. Since then, these oil and gas companies have pursued further exploration and development, throughout which the potential for increased underwater sounds and vessel traffic to disturb and displace gray whales has been of great concern.

To develop mitigation measures intended to minimize impacts to gray whales in their feeding areas, the two main oil and gas companies operating in the region, ENL and SEIC, partnered in a western gray whale research program. This program, which has been ongoing since 2002, focuses primarily on life history aspects of western gray whales and their acoustic environment while in the Sakhalin feeding areas (e.g., Blanchard et al., 2019; Demchenko, 2007, 2010; Demchenko et al., 2016; Durkina et al., 2017; Fadeev, 2011; Gailey et al., 2009; Gritsenko et al., 2014; Mate et al., 2015; Tyurneva et al., 2010, 2012; Yakovlev et al., 2009, 2011). Data from this program have been instrumental in mitigation planning (Bröker et al., 2015; Johnson et al., 2007).

ENL is the operator of the Sakhalin-1 fields Odoptu, Chayvo, and Arkutun-Dagi (Fig. 1). The Odoptu field is located adjacent to the northern part of the nearshore feeding area and its reserves are accessed from onshore drilling facilities. The Chayvo field is located towards the southern end of the nearshore feeding area and has been developed using both onshore and offshore drilling facilities. The Arkutun-Dagi field is located 25 km offshore, eastward of Chayvo Bay, just north of the offshore feeding area. ENL planned seismic surveys in these three fields during the summer of 2015, using three seismic vessels. SEIC, operator of the Piltun-Astokh field that is located between ENL’s Odoptu and Chayvo fields, also planned to conduct a seismic survey in 2015, using a single seismic vessel. These seismic vessels tow an array of air guns and seismic streamers (consisting of cables to which hydrophones are attached); both the array and streamers are towed a few meters below the water surface. The air guns in the array use compressed air and, collectively, produce downward focused sound waves capable of penetrating underlying geological features, which both refract and reflect sound waves back toward the surface. The streamer hydrophones detect the returning waves and record their amplitude and travel time to the various subsurface layers. The return times of the sound waves provide information about the location and quantity of gases or liquids in the subsurface layers. Seismic vessels typically travel at speeds in the range of 4 to 5 knots along a pattern of parallel lines covering the survey area, with air guns releasing compressed air at preset intervals of approximately 7 to 8 s.

The planned operations for 2015 consisted of more seismic survey activity near the gray whale feeding areas than was known to have occurred in the previous two decades. Disturbance of gray whale feeding activity was of great concern, especially in the nearshore feeding area, the only location near Sakhalin where mother-calf pairs have been observed (Tyurneva et al., 2018).

Analyses of data collected during previous seismic surveys along the northeast Sakhalin coast have identified responses to survey sounds including increases in swimming speed, changes in distance from shore, changes in dive times, and avoidance of the area (Gailey et al., 2007; Yazvenko et al., 2007a, b). While a disturbance may cause statistically significant changes in behavior it is unclear how such changes affect population growth, but there is the possibility that many seemingly small cumulative disturbances from multiple stressors can lead to population decline. Population growth is therefore the ultimate metric with which species and populations are managed. Conceptual models, such as the Population Consequences of Disturbance (PCoD) models, have been developed and modified over many years to establish a framework to quantify population-level impacts from anthropogenic and other types of disturbances (NAS, 2017; New et al., 2014; NRC, 2005).

In addition to the development of a mitigation program intended to reduce disturbance to gray whales due to the 2015 seismic surveys, studies were implemented to assess the effectiveness of the mitigation measures and to inform bioenergetics models that predict population-level effects of reduced foraging due to acoustic exposure. More specifically, the data collection protocols focused on informing stochastic dynamic programming (SDP) bioenergetics models that examined how reduced foraging at the individual level due to acoustic disturbance might affect reproduction and survival of pregnant females. That is, for the first time in this region, studies were designed to quantify the functions and to ground truth the models that connect behavioral changes with demographic rates, particularly within the context of prey availability.

The first part of this paper summarizes the mitigation approach, the second part summarizes the implementation of the mitigation measures during ENL’s seismic surveys, and the third part summarizes the overall strategy behind assessment of impacts and mitigation effectiveness. Results of this assessment, including western gray whale behavior and distribution responses to the 2015 seismic survey activities, availability of prey resources, and how a potential decrease of energy intake due to acoustic disturbance could affect reproductive success, are presented in separate papers within this special issue (Blanchard et al., 2022a, b; Gailey et al., 2022a, b; Maresh et al., 2022; Rutenko et al., 2022a, b; Schwarz et al., 2022a, b). It should be noted that ENL and SEIC implemented comparable but not identical mitigation measures; SEIC’s approach is summarized and discussed in IUCN (2015a, b, c, 2016) and is not part of this paper.

## Part I: Mitigation approach

### Early survey completion

The most efficient measure to reduce impacts on marine mammals due to sound exposure from seismic surveys is to conduct the activity at a time during which no or few animals would be present (Nowacek et al., 2013). The primary mitigation principle in 2015 prioritized early completion of the seismic surveys closest to the nearshore feeding area, when fewer gray whales would be present due to their migration timing. A similar approach was applied to a seismic survey near Sakhalin Island in 2010 (Bröker et al., 2015).

Sea ice conditions dictated the earliest possible start date, but once conditions allowed work to proceed early completion relied on operational efficiency. ENL therefore planned to use two seismic vessels for the Odoptu survey, closest to the nearshore feeding area, with one seismic vessel collecting data on one survey line while the other vessel was repositioning itself to collect data on another survey line. At the same time, a third seismic vessel was operating in the Chayvo survey area. To further minimize survey delays, implementation of air gun shutdown measures intended to minimize disturbance to feeding gray whales would not commence until later in the season (see seasonal criteria below). The assumption was that allowing disturbance of fewer whales early in the season would have less impact on the population than extending the survey duration to later in the season, when more gray whales would have arrived. Arguably, due to the phased migration of gray whales by age or sex class and reproductive status, this mitigation approach could have increased exposure of individuals that typically arrive on the feeding grounds early, such as reproductive females.

### Establishment of mitigation zones

To minimize disturbance to gray whales in the nearshore feeding area, ENL implemented air gun shutdown procedures for animals that would be exposed to broadband sound levels ≥ 163 dB re 1 μPa^2^ root mean square sound pressure level (SPL^1^) (Table 1). This sound level threshold is based on an air gun sound playback study in the Bering Sea during the 1980s, which estimated that 10% of gray whales stopped feeding and moved away from the area when exposed to received air gun sounds of that level (Malme et al., 1988). This criterion was also used during other seismic surveys near Sakhalin Island in 2001 and 2010 (Bröker et al., 2015; Johnson et al., 2007).

**Table 1:** Summary of air gun shutdown and ramp up procedures implemented during the 2015 Odoptu, Chayvo, and Arkutun-Dagi (AD) seismic surveys

Procedures	Description
Air gun shutdown to minimize gray whale disturbance	• Air guns were shut down when gray whales in the nearshore feeding area were observed to enter the 163 dB re 1µPa^2^ SPL footprint of active seismic vessels. These shutdowns were implemented after 1 July for survey lines in Zone A1 and 15 July for survey lines in Zone A2. • Operations were allowed to resume when the gray whale was seen outside the 163 dB re 1µPa^2^ SPL footprint or 20 min after the shutdown occurred and no gray whales were seen. • Poor visibility measures applied when conditions did not allow reliable detection of gray whales in the 163 dB re 1µPa^2^ SPL footprint of a line in Zone A1 or A2.
Air gun shutdown to prevent auditory injury	• Modeled injury threshold distances for cetaceans were ≤ 100 m for all seismic vessel locations and species hearing sensitivities considered; for otariid pinnipeds the injury thresholds were not reached (Matthew et al., 2018). ENL adopted a 500-m safety zone distance for cetaceans and 50 m for the endangered Steller sea lion (*Eumetopias jubatus*). • After MMOs onboard the seismic vessels determined that no cetaceans and Steller sea lions were observed within their applicable safety zones, ramp up of air guns was initiated. • Once ramp up started, air guns were shut down only when endangered or threatened cetaceans species – western gray whale (*Eschrichtius robustus*), Okhotsk population of bowhead whales (*Balaena mysticetus*), North-Pacific right whale (*Eubalaena japonica*), fin whale (*Balaenoptera physalus*), and sei whale (*Balaenoptera borealis*) – were observed within or entering the 500-m safety zone or when Steller sea lions were observed within or entering the 50-m safety zone. • Air gun ramp up following a shutdown was initiated after the animal was observed to leave the area or 20 min after shutdown occurred (assuming no other cetaceans were observed within the 500-m safety zone or no Steller sea lions were observed in the 50-m safety zone). • When conditions did not allow the reliable detection of marine mammals within the 500-m safety zone, poor visibility measures applied.
Air gun ramp up	• Ramp up involved activating a progressively larger number of air guns over a 20 min period. • Ramp up was required after air gun silence of 20 min or more. • Prior to initiating a ramp up, the 500-m safety zone had to be clear of marine mammals for a period of 20 min.

During the planning phase, various seismic array configurations were investigated for possible reduction of the size of the 163 dB re 1 µPa^2^ SPL isopleth while maintaining the quality of the seismic imaging. This yielded a configuration of 24 air guns in two strings, ranging in volumes from 45 to 290 in^3^, for a total of 2340 in^3^ for each of the three seismic vessels (Rutenko et al., 2022a).

To facilitate prioritization of operations along seismic survey lines having the highest spatial overlap with the nearshore feeding area, the Odoptu and Chayvo seismic survey areas were divided into mitigation zones based on the 163 dB re 1 μPa^2^ SPL footprint of the full air gun array. The 163 dB re 1 μPa^2^ SPL footprint of each seismic survey line was estimated through pre-survey acoustic modeling that was calibrated and adjusted during the survey using real-time acoustic data (Rutenko et al., 2022a). The boundary of the nearshore feeding area, for mitigation purposes, was defined as the 95% kernel of the gray whale density surfaces (whales/km^2^) using gray whale sighting data from scans at shore-based locations and line transect surveys (vessel and aerial) from June and July 2002–2013 (Fig. 1). The methodology for estimating the density surface and calculating the 95% kernel contour is described in Muir et al. (2015). The area outside the kernel contour was not considered to be part of the nearshore feeding area.

The mitigation zones were also defined based on gray whale distribution within the nearshore feeding area. Gray whale densities have been consistently higher nearshore of the 20 m contour (Vladimirov et al., 2013), and mother-calf pairs predominantly occur at distances less than 1 km from shore in waters shallower than 10 m (Sychenko, 2011). In some years, aggregations of gray whales were observed beyond the 20-m contour, which may be associated with increased sand lance (*Ammodytes hexapterus*) abundance in the area (Fadeev, 2011; Kriksunov et al., 2016).

Ultimately, three mitigation zones were established. Zone A1 encompassed data acquisition lines for which the 163 dB re 1µPa^2^ SPL footprint would reach portions of the nearshore feeding area ≤ 20 m deep. Zone A2 encompassed data acquisition lines for which the 163 dB re 1µPa^2^ SPL footprint would only reach portions of the nearshore feeding area > 20 m deep. Zone B encompassed data acquisition lines for which the 163 dB re 1µPa^2^ SPL footprint would not reach the nearshore feeding area. The Odoptu survey area comprised all three zones, the Chayvo survey area only Zones A1 and B, and the Arkutun-Dagi area only Zone B (insets in Fig. 1).

### Seasonal criteria to reduce disturbance

**Fig. 2:**
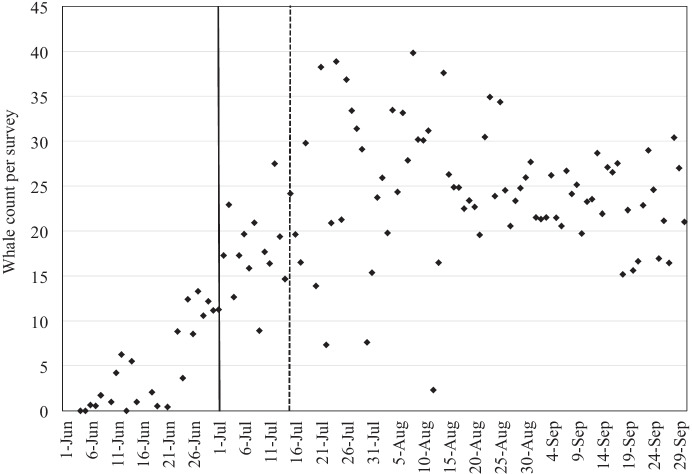
Seasonal gray whale abundances within the nearshore feeding area based on scan data from aerial (2001–2005), vessel-based (2002–2013), and shore-based (2004–2013) surveys were used to establish seasonal criteria for behavioral shutdowns: 1 July for Zone A1 (solid line) and 15 July for Zone A2 (dotted line). Whale count per survey is the average number of whales counted per survey per day

Seasonal criteria were established for lines in Zone A1 and A2. Although whales are present in the nearshore feeding area in early June, there is an indication that mother-calf pairs begin to arrive sometime around the end of June (Gailey et al., 2016). Furthermore, visual examination of scan data suggests that the increase in number of gray whales plateaus around mid-July (Fig. 2).

Without information on arrival and departure times of individuals, we assumed the inflection point in mid-July indicated arrival of most whales. With this in mind, the mitigation approach allowed continuation of seismic survey operations during June without implementation of mitigation measures to reduce disturbance of gray whales in the feeding area. Starting 1 July for operations in Zone A1 and 15 July for operations in Zone A2, the mitigation plan required air guns to be shut down when observed gray whales in the nearshore feeding area would likely be exposed to sound levels ≥ 163 dB re 1µPa^2^ SPL based on acoustic footprints. Air gun shutdown procedures to reduce disturbance to gray whales were not implemented for operations in Zone B, since the 163 dB re 1µPa^2^ SPL footprint of these lines would not reach the nearshore feeding area. A real-time data collection and transfer system was developed to aid in the implementation of these behavioral shutdowns.

### Measures for preventing auditory injury

Air gun sounds have the potential to cause auditory injury in nearby marine mammals. Manifestations of auditory injury, such as temporary and permanent threshold shifts, are not detectable in the field and have therefore mostly been studied in laboratories for odontocete and pinniped species (review in Southall et al., 2007). No direct measurements exist for mysticetes. A common mitigation practice to prevent auditory injury has been the establishment of a buffer or safety zone around the air gun array, either by using a radius to specific received sound levels or a fixed distance (Weir & Dolman, 2007). ENL used the most recent scientifically based injury criteria (Finneran & Jenkins, 2012; Southall et al., 2007) to estimate, using acoustic models, the distance at which auditory injury could occur for endangered marine mammal species known to be present in the area. These modeling results were reviewed and verified before a subsequent seismic survey when updated scientific injury criteria and a more advanced version of the acoustic source model were applied (Matthews et al., 2018). Although in all modeled cases the results indicated that the injury threshold distances were < 500 m, a 500-m safety zone was adopted (Table 1).

During all seasons and in all zones, ENL implemented air gun shutdown and gradual ramp up procedures for endangered or threatened cetaceans seen within or entering the 500-m zone around air guns to avoid exposing them to sound pulses that could cause auditory injury (Table 1). Two marine mammal observers aboard each of the three seismic vessels were on watch during daylight hours and were responsible for monitoring the safety zone and for implementing safety zone shutdowns according to these procedures.

### Poor visibility conditions

Whales cannot be observed in darkness or during periods of dense fog or heavy precipitation. The period of darkness varies throughout the season, with a total duration of about 5 h per day in June to about 12 h per day in September. Foggy conditions occur frequently and unpredictably along the northeastern Sakhalin coast, especially in July and August. Taking into account the primary mitigation goal of completing the seismic surveys as early in the season as possible (when the smallest numbers of gray whales are present), operations during periods of darkness and fog were allowed under certain circumstances. In Odoptu Zone A1 or A2 or in Chayvo Zone A1, starting a line during visibility conditions insufficient to detect gray whales in the nearshore feeding area would be allowed if no behavioral shutdowns for gray whales had occurred during a preceding period of 24 h of good visibility. During this preceding period, the 163 dB re 1µPa^2^ SPL footprint inside the nearshore feeding area had to be visible for detection of gray whales. The same poor visibility criteria applied to safety zone mitigation procedures intended to prevent auditory injury. Operations along any line would only be allowed during poor visibility conditions if no safety zone shutdown had occurred in these zones during a preceding 24-h period where the 500-m safety zone would have been visible.

## Part II: Mitigation implementation

### Seismic data acquisition

In 2015, ENL conducted seismic surveys in the Odoptu, Chayvo, and Arkutun-Dagi license areas (Fig. 1). In Odoptu two seismic vessels (M/V *Polarcus Amani* and M/V *Polarcus Asima*) were used. In Chayvo, where water depth was too shallow for the large Polarcus vessels to navigate safely, the M/V *Orient Explorer,* a seismic vessel with shallower draft and equipped with fewer streamers, was used. Seismic data acquisition in the Odoptu and Chayvo survey areas started on 11 June, which was as soon as ice conditions allowed. The data acquisition in Odoptu was completed in 28 days; Zone A1 lines were completed on 30 June, Zone A2 lines on 1 July, and Zone B lines on 7 July. The two Polarcus vessels moved to the Arkutun-Dagi survey area on 8 July to continue seismic data acquisition there until 23 September. The M/V *Orient Explorer* completed all Zone A lines of the Chayvo survey area on 27 July. SEIC’s 2015 Piltun-Astokh seismic survey, utilizing one seismic vessel, started seismic data acquisition on 8 July, immediately after ENL completed the survey in Odoptu. Total duration of the Piltun-Astokh seismic survey was 21 days (IUCN, 2016).

In Odoptu and Chayvo, the three seismic vessels spent ~ 50% of the time on production, meaning the vessels were traveling along a line while the full air gun array was active or transferring to the next line, during which a single air gun usually remained active (Table 2). The large proportion of time spent on mobilization, especially for the two Polarcus vessels (36.3% and 39.0%), reflects the initial stand-by period waiting for ice to have sufficiently cleared to start operations. Delays due to tide and current ranged from 5.5 to 12.4% of the time. Delays due to implementation of safety or behavioral zone air gun shutdowns ranged from 0.1 to 0.4%. The larger proportion of delay due to poor visibility procedures that occurred during M/V *Polarcus Asima* operations (4.6%) compared to the other two seismic vessels was related to the 24-h reset rule after a safety shutdown, which happened during a period of persistent fog.

**Table 2:** Seismic survey activities of the three ENL seismic vessels while operating in the Odoptu survey area (M/V *Polarcus Asima* and M/V *Polarcus Amani*) and Chayvo survey area (M/V *Orient Explorer*)

Activity Category	*Polarcus Asima*		*Polarcus Amani*		*Orient Explorer*	
	hours	%	hours	%	hours	%
Mobilization period (includes tests)	348.6	36.3	396.2	39.0	235.3	17.1
Production (full source active)	184.8	19.2	204.0	20.1	325.7	23.7
Line changes	267.0	27.8	337.7	33.2	382.2	27.8
Fisheries restrictions	n/a	n/a	n/a	n/a	9.8	0.7
Cetacean delays (ramp up, shutdown)	0.5	0.1	1.0	0.1	5.5	0.4
Poor visibility delays	43.9	4.6	3.5	0.3	14.8	1.1
Current and tide delays	74.9	7.8	56.3	5.5	170.3	12.4
Weather standby	0.9	0.1	0.0	0.0	5.6	0.4
Other delay (technical, field ops)	35.4	3.7	9.3	0.9	178.4	13.0
Transit to Arkutun Dagi	4.9	0.5	8.7	0.9	n/a	n/a
Demobilization	n/a	n/a	n/a	n/a	46.7	3.4
Total	960.8		1016.7		1374.3	

### Real-time data collection and transfer

The newly developed ability to centralize all gray whale location and acoustic data in real-time, which required advanced data telemetry and customized networking software, proved to be an important improvement over previous mitigation approaches (Bröker et al., 2015; Johnson et al., 2007). A central base station near Odoptu was set up (referred to as Central Post) where a team of scientists received and interpreted real-time information from field teams and acoustic recorders.

Five shore-based teams used theodolites to track movements of gray whales within the nearshore feeding area that were near or approaching the 163 dB re 1 µPa^2^ SPL footprint (Fig. 1). These teams used a custom version of Pythagoras (Gailey & Ortega-Ortiz, 2002) that calculated and transmitted animal positions via cellular modems to the Central Post computer, which also used the Pythagoras GIS command window to display Automatic Identification System (AIS) data from vessels, gray whale sighting data, and acoustic data from the seismic vessels in real-time.

**Fig. 3:**
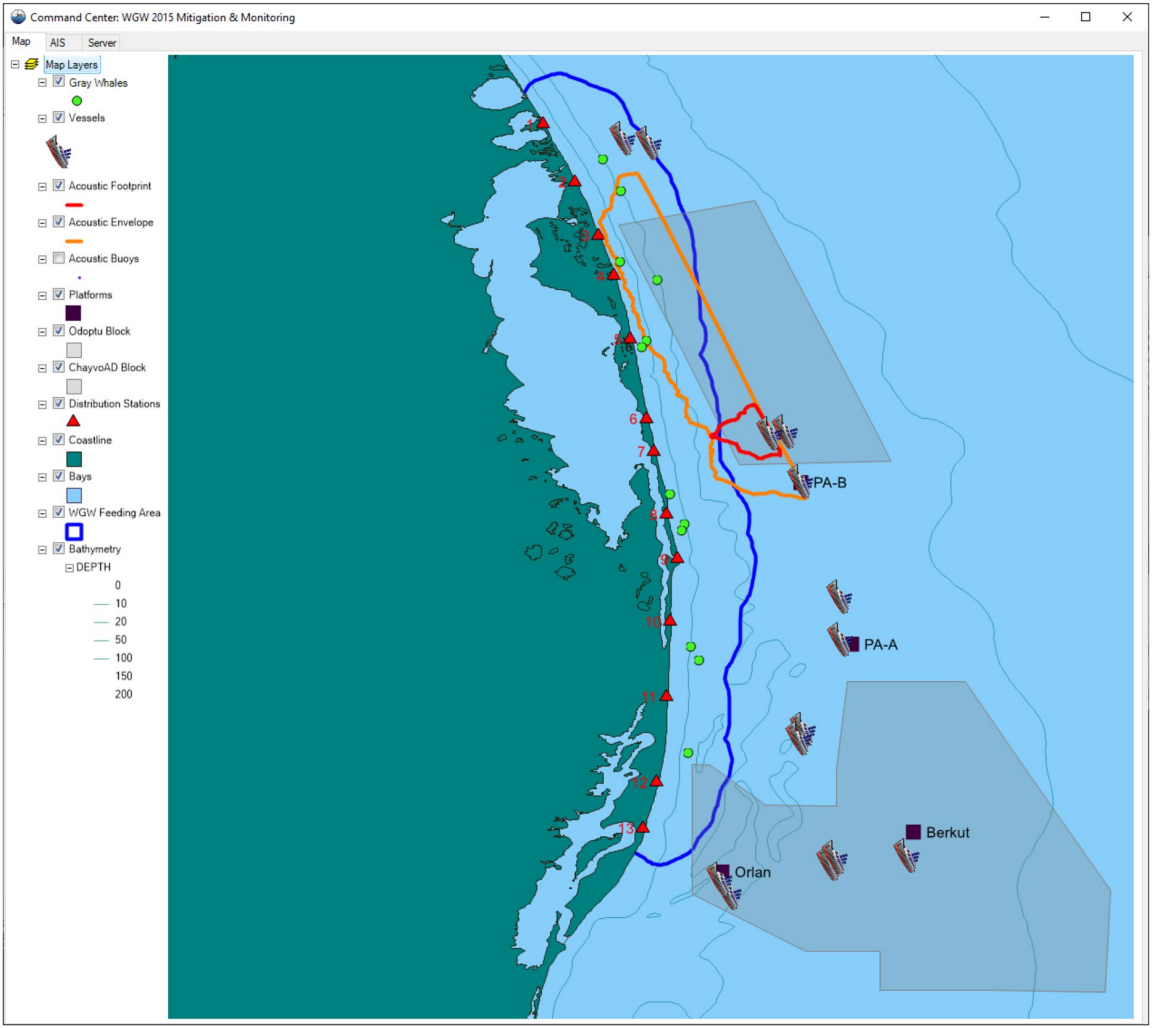
Integrated real-time processed acoustic and gray whale location data and vessel positions as received by the Central Post. The 95% kernel contour (blue line) represents the nearshore feeding area boundary. The pre-season modeled and field adjusted 163 dB re 1 µPa^2^ SPL footprints were displayed for both the entire survey line (orange) and for the active air gun array progressing with the moving seismic vessel (red). Gray whale location data from shore-based teams are shown as green dots

Ten Autonomous Underwater Acoustic Recorders with VHF and Iridium data transmission capability (RI-AUAR) were deployed along the 20-m isobath near the Odoptu (up to 4) and Chayvo (up to 6) seismic survey areas during operations (Fig. 1). Data were telemetered to the Central Post by two complementary methods: through digital VHF radio transmission of the acoustic waveform to three receiving stations on shore that analyzed the data and relayed sound levels to the Central Post, and through processing of the acoustic waveform within the RI-AUAR and transmission of sound levels via Iridium satellites. The real-time acoustic data were used to calibrate pre-season modeled 163 dB re 1 µPa^2^ SPL footprints by providing a measure of the discrepancy between model predictions and measured levels at the RI-AUARs, from which acousticians stationed at the Central Post estimated a correction factor and applied it to the modeled levels defining the footprint (Rutenko et al., 2022a). The calibrated 163 dB re 1 µPa^2^ SPL footprint of the entire line (the acoustic envelope) and the footprint associated with the air gun array, which moved with the seismic vessel as it progressed along the data acquisition line (the acoustic footprint), were transmitted to the Pythagoras software for display (Fig. 3). In this manner, staff in the Central Post could observe on an overlay map both the acoustic footprint moving with the vessel and the gray whale sighting locations, along with other key situational information.

Starting 1 July for Zone A1 and 15 July for Zone A2 operations, Central Post personnel gave seismic vessels a 20 to 30 min pre-warning if it appeared likely, based on observed gray whale locations and movement data as well as vessel speed and course, that a gray whale would be exposed to sounds of 163 dB re 1 µPa^2^ SPL or greater. Just before the gray whale would have been exposed to 163 dB re 1 µPa^2^ SPL, based on available information, the seismic vessel would be instructed to shut down the air guns.

### Air gun shutdowns

A total of nine air gun shutdowns were implemented from all three seismic vessels during ENL’s seismic surveys, four of which were implemented to limit behavioral responses and five of which were implemented to prevent potential auditory injury. The four shutdowns implemented to limit behavioral responses occurred during the Chayvo survey after 1 July. Data acquisition of lines in Zone A1 and A2 of the Odoptu survey were completed before the behavioral shutdown criteria became active. The five shutdowns implemented to prevent potential auditory injury were triggered by gray whales sighted within or entering the 500-m safety zone. Observers on the seismic vessels recorded 874 cetacean sightings during 4779 observer hours accumulated from 11 June to 23 September 2015 (Table 3). Gray whales represented 36% (314) of all sightings, including the five sightings within or entering the 500-m safety zone during seismic data acquisition. Steller sea lions were not observed within the 50-m safety zone defined for this species. Non-endangered cetaceans sighted within 500 m included eight minke whales (*Balaenoptera acutorostrata*), four killer whales (*Orcinus orca*), one Dall’s porpoise (*Phocoenoides dalli*), and two harbour porpoises (*Phocoena phocoena*). Safety zone shutdowns for non-endangered cetaceans were not required after air gun ramp up was initiated.

**Table 3:** Marine mammal sighting summary from observers aboard the three seismic vessels. Observer effort was 1914 h for M/V *Polarcus Asima*, 1852 h for M/V *Polarcus Amani*, and 1013 h for M/V *Orient Explorer*. Reduced source refers to situations when a subset of air guns was active (e.g., during ramp up or line changes)

Closest observed distance to source	Gray whales	Other endangered/threatened cetaceans	Non-endangered/threatened cetaceans	Total cetacean sightings	Steller sea lions
< 500 m					
Full source	5*	0	15	20	1
Reduced source	2	2	30	34	8
No source	7	12	80	99	4
> 500—1000 m					
Full source	14	0	38	52	2
Reduced source	11	1	47	59	0
No source	20	15	73	108	1
> 1000—2000 m					
Full source	8	0	34	42	0
Reduced source	17	0	41	58	0
No source	25	10	51	86	0
> 2000 m					
Full source	68	0	22	90	0
Reduced source	79	3	30	112	0
No source	58	12	26	96	0
Unknown source	0	3	15	18	2
Total	314	58	502	874	18

*Indicates sightings for which air gun safety zone shutdowns were implemented

## Part III: Strategy to assess impacts and mitigation effectiveness

### Assessment framework

While a rational approach to mitigating potential impacts from anthropogenic sounds is an important component of many oil and gas industry seismic programs, the efficacy of mitigation is seldom evaluated. Parts I and II of this paper described the approach to mitigation during 2015 and the implementation of the mitigation measures. Here, the overall strategy behind the investigation of impacts and mitigation effectiveness is described.

With the prior knowledge that gray whales in the nearshore feeding area will display changes in behavior and distribution due to sound exposure and proximity of vessels during the 2015 seismic survey activities, several hypotheses were developed to determine how these responses would affect foraging success. This is especially important for small marine mammal populations with high site fidelity that might not have alternative feeding areas (Forney et al., 2017), such as the western gray whales.

The effectiveness of the mitigation measure specifically designed to reduce disturbance to gray whales in the nearshore feeding area (behavioral shutdowns) was assessed through multi-variate analyses documenting changes in gray whale behavior and distribution related to sound exposure and proximity of vessels (Gailey et al., 2022a, b). These analyses have been done before, but either lacked information on received sound levels at whale locations (Gailey et al., 2007; Yazvenko et al., 2007a) or did not have sufficient sample size to detect subtle-to-moderate changes in behavior or distribution (Gailey et al., 2016; Muir et al., 2015).

A crucial component of the mitigation assessment strategy in 2015, implemented for the first time in this region, involved the development of a bioenergetics model (McHuron et al., 2021; Schwarz et al., 2022b). The model framework incorporated knowledge of the energetic needs of gray whales for successful reproduction and survival, evidencing the relationship between lost foraging opportunities and reduced population growth (Villegas-Amtmann et al., 2015, 2017). SDP bioenergetics models (Clark & Mangel, 2000; Houston et al., 2006; Mangel & Clark, 1988; Mangel & Ludwig, 1992) were subsequently applied, accounting for behavioral adaptations that gray whales may use to maximize energetic intake in the face of disturbance (McHuron et al., 2021). The bioenergetics model developed for the Sakhalin area, in other words, accounts for gray whale movement to—and foraging in—other areas when they are disturbed, rather than assuming the whales have no way to compensate for lost foraging opportunities. Through the ability to simulate various disturbance scenarios and allow individuals to vary their behavioral responses, the SDP bioenergetics models can mimic natural situations. These models require energetics, behavior, and prey availability data of the species of interest as well as data about the disturbance itself.

Results from the multi-year western gray whale research program combined with the anticipated level of disturbance in 2015 provided an initial framework to better understand the consequences of acoustic disturbance relative to documented behavioral and distribution responses (McHuron et al., 2021). With this framework in mind, ENL improved the design of the studies in 2015 to better inform SDP bioenergetics models. These studies, intended to document how changes in gray whale distribution and behavior as a response to acoustic disturbance from seismic survey activities might affect energy intake, compiled 1) acoustic data, thereby documenting potential causes of disturbance; 2) prey biomass and energy density based on benthic sampling, thereby documenting prey availability; and 3) observations of gray whale distribution and behavior. In addition, whales were photographed as part of a long-term effort to better understand population trends. The data from the photo-identification studies, the distribution studies, and behavior studies could be used to ground-truth SDP bioenergetics model results. Each of these four components of the program is described below.

### Acoustic data

Analyzing behavior responses of gray whales and possible changes in distribution due to seismic survey activities requires knowledge of the acoustic environment. The acoustic program was designed to characterize the acoustic field inside and outside of the nearshore feeding area from all known anthropogenic sound sources during the 2015 season (seismic, pile driving, shipping, fishing, and production operations). To accomplish this monitoring objective 40 AUARs were deployed, covering 48 different monitoring locations throughout the season, resulting in 126 AUAR deployments for 4312 recorder days over a 142-day period of acoustic measurements. From these data, the noise fields of various anthropogenic activities were estimated using acoustic propagation models calibrated with measured levels from the AUARs (Rutenko et al., 2022b). Using these calibrated propagation models, various acoustic metrics were calculated for observed whale tracks and for a 1-km^2^ grid that covered the viewing area of the shore-based observers. These metrics were used in gray whale behavior and distribution analyses (Gailey et al., 2022a, b) and in the SDP bioenergetics models (Schwarz et al., 2022b). In contrast to the behavior and distribution analyses, which only focused on the nearshore feeding area, the bioenergetics model also investigated potential disturbance of feeding gray whales that might have moved from the nearshore to the offshore feeding area. The estimation of acoustic variables for the offshore feeding area was performed for a coarser 100-km^2^ grid (Rutenko et al., 2022b), mainly because there was only one AUAR in the offshore area recording acoustic levels that could be used for model calibration. This was adequate considering the resolution of gray whale distribution and benthic data in the offshore feeding area required for the SDP model.

### Prey biomass and energy density

The design of the benthic study was formulated around knowledge of the various scenarios of gray whale displacement due to the 2015 seismic survey activities and the need to understand availability of prey resources following displacement. Three approaches to sampling were used (Blanchard et al., 2019, 2022a): 1) A dense sampling grid of 108 locations covering the nearshore feeding area where, historically, whale density was highest; 2) Targeted sampling at locations where gray whales were observed to be feeding; and 3) Repeat sampling at locations within nearshore and offshore feeding area grid cells from which benthic samples had been collected in 2002–2014.

Benthic samples were collected three times during the field season within the dense grid to examine spatial and seasonal variability of prey resources. Prey biomass values of the samples collected at locations where gray whale feeding activity had been observed were compared to those of the dense grid samples. The repeat sampling of the “historic” grid provided an indication of the prey availability in both feeding areas in relation to previous years and also served to document the long-term trend in prey availability (Blanchard et al., 2019). In addition, estimates of caloric content of main prey species were derived from selected samples taken in the nearshore and offshore feeding areas (Maresh et al., 2022), and spatial interpolation of prey data (Blanchard et al., 2022b) provided prey variables for input into the gray whale behavior, distribution, and bioenergetics models.

### Gray whale distribution and behavior

Large sample sizes are required to account for natural variability in abundance, distribution, and behavior of gray whales as well as to assess subtle-to-moderate responses to various activities (Dunlop et al., 2018; Gailey et al., 2016). With mitigation measures designed to minimize behavioral responses, coupled with relatively short-duration seismic surveys conducted when fewer gray whales are in the area, the documentation of measureable responses becomes difficult (Gailey et al., 2016; Muir et al., 2016; Muir et al., 2015). Obtaining sufficient sample sizes needed to detect subtle-to-moderate behavioral changes, as documented by Gailey et al. (2016), called for an increased monitoring effort. As such, shore-based teams, each with at least two observers at any time, were based at 13 gray whale monitoring stations along the entire nearshore feeding area from June through September. Using scan sampling techniques, the teams conducted hourly scans for gray whales, weather permitting. During a collective total of 1277 observer days, 10042 scans were completed (Gailey et al., 2022b), which was about 1.5 times the total combined number of scans collected across 14 years of gray whale distribution data. Since the shore-based observers only covered the nearshore feeding area, two vessel-based observers monitored gray whales along line transects in the offshore feeding area. Frequency of line transect sampling was dependent on weather conditions and vessel availability but was targeted at twice a month. A total of nine line transect surveys were completed from June through October, with gray whale counts ranging from a minimum of 22 (end of June) to a maximum of 83 (early September.)

In addition to hourly scans, at 8 of the 13 stations, chosen because they were closest to seismic operations, observers with theodolites collected detailed data on gray whale movements and respiration (Gailey et al., 2022a). These observers recorded a total of 1270 gray whale tracks covering ~ 1140 h and 401 focal follows of ~ 440 h, which more than quadrupled the amount of movement and respiration data collected during three previous seismic surveys combined. The relationship between gray whale responses, various natural variables including prey biomass, and seismic survey activities were quantified using multivariate analyses (Gailey et al., 2022a, b).

### Photo-identification data

Photo-identification data are useful to better understand population level responses because they provide insight into individual preferences for foraging in the nearshore or offshore feeding areas (Schwarz et al., 2022a), body condition of reproductive females, reproductive success, and population trends; such data are thus useful for verifying results of bioenergetics model simulations (Schwarz et al., 2022b). During the 2015 season a total of nine teams were collecting photo-identification data, increasing the likelihood of photographing all whales present during the foraging season and maximizing daily and seasonal resighting rates of individual gray whales. Five of those teams specifically focused on photographing whales that were tracked and for which respiration data were collected. This information is valuable for determining how individuals might respond differently to acoustic exposure or might be exposed repeatedly. Two of the nine teams were traveling up and down the coast, augmenting photographic data collected by the five behavior teams. The remaining two teams were vessel-based, with one operating in the nearshore feeding area from a small boat launched from shore and the other operating predominantly in the offshore feeding area from a research vessel or from a small boat deployed from that vessel. Due to the increased photo-identification effort in 2015, resighting rates across the season were the highest recorded to date. Ninety-four percent of individuals were seen on more than one day in 2015, with one whale seen on 42 days (Schwarz et al., 2022a).

## Summary

The real-time collection and telemetry of acoustic and whale sighting data to a centralized location, overlaid with seismic vessel positions, were instrumental for identifying situations where whales might be exposed to 163 dB re 1μPa^2^ SPL and for efficient implementation of air gun shutdowns as per the mitigation criteria. This approach, largely possible due to technological advances, was an improvement to that used in the 2001 seismic survey, during which a static distance of 4 km from the seismic vessel, based on the 163 dB re 1μPa^2^ SPL criteria, was used for behavioral shutdown decisions (Johnson et al., 2007). The likelihood of gray whale presence within this zone was determined through aerial surveys 2–4 h prior to initiating seismic data acquisition in Zone A. Similarly, the approach described here, used in 2015, stands in contrast to the approach used in 2010, when a static acoustic footprint was calculated for each line of the seismic survey and behavioral shutdowns were implemented for gray whales within the entire footprint, regardless of seismic vessel position (Bröker et al., 2015). This approach resulted in shutdowns for animals exposed to sound levels much lower than the 163 dB re 1μPa^2^ SPL criteria, introducing operational delays. In short, the approach and implementation of air gun shutdown measures in 2015, in contrast to earlier surveys, improved operational efficiencies and thereby decreased the duration of activities with the potential to impact marine mammals.

The 2015 mitigation approach described in this paper allowed, by design and for the first time since the 2001 survey, exposure of western gray whales to sound levels above 163 dB re 1 µPa^2^ SPL, with the goal of completing seismic survey operations as early in the season as possible. These higher exposures occurred predominantly in June, before the majority of gray whales—and particularly mother-calf-pairs—arrived. Despite this change, observed exposures to sound levels higher than 163 dB re 1 µPa^2^ SPL were infrequent (Gailey et al., 2022a).

In conjunction with mitigation efforts, data collection strategies designed with the PCoD conceptual framework in mind were implemented in an effort to assess efficacy of mitigation. As such, the data collection approach was not only designed to understand the acoustic environment, prey resources, gray whale behavior, and habitat use, but also to identify gray whale responses to seismic survey activities. Details about the nature of these responses and how the responses related to the goal of minimizing disturbance to gray whales on their feeding ground are described in Gailey et al. (2022a, b).

The impact assessment strategy went beyond the conceptual PCoD model with the development of SDP bioenergetics models to predict how different levels of acoustic disturbance could affect feeding activity of pregnant females and how that might influence reproductive success. Initial model results indicated that availability of high-energy prey resources appeared to be a major factor determining pregnant female and calf survival in the face of disturbance (McHuron et al., 2021). Model simulations also illustrated that acoustic disturbance early in the season would be potentially less harmful than later in the season, based on hypothetical disturbance scenarios that considered seismic surveys to be the sole source of disturbance and considering the available prey resources reported for 2015 (McHuron et al., 2021). With this newly-developed SDP bioenergetics model framework for western gray whales, an additional model simulation was run using the acoustic data from the 2015 seismic survey activities as the disturbance scenario (Schwarz et al., 2022b). Habitat use patterns from the 2015 distribution data and reproductive success from photo-identification data were used to validate the model.

Since gray whales are exposed to multiple disturbances when migrating between feeding and breeding grounds, the intent is to expand the bioenergetics model framework with multiple foraging seasons and other reproductive states to predict population level impacts from cumulative multi-year exposures using dose–response associations for western gray whales on their feeding grounds. Photo-identification data from 2015 and subsequent years (e.g., Tyurneva et al., 2018) are useful to monitor the population status and reproductive rates and to identify individuals or groups of individuals that may be more sensitive to anthropogenic activities. Photo-identification data can also validate SDP bioenergetics model predictions, since these data provide information on the number of pregnant females during the year of disturbance, the number of females observed on the feeding grounds with calves during subsequent years after the disturbance, and the number of calves observed in the future (Schwarz et al., 2022b). Although models will not capture all of the complexities of disturbance and animal behavior (McHuron et al., 2021), the SDP bioenergetics model framework offers a useful tool for examining potential biologically significant impacts on western gray whales due to anthropogenic disturbances and assists in developing appropriate mitigation measures to minimize acoustic disturbance.

## Footnotes

1 The definition of the SPL metric as mean square pressure level with reference value 1 µPa^2^ conforms with the acoustical terminology standards ISO 18405 and ANSI S1.1, and adheres to the original definition of the decibel in terms of a logarithmic ratio of power quantities. Earlier literature may refer equivalently to root mean square (rms) pressure level with reference value 1 µPa.
